# High-intensity chemotherapy improved the prognosis of patients with high-grade B-cell lymphoma

**DOI:** 10.3389/fimmu.2022.1047115

**Published:** 2022-12-23

**Authors:** Yanfang Chen, Qing Cai, Yu Chang, Mingzhi Zhang, Zhaoming Li

**Affiliations:** ^1^ Department of Oncology, the First Affiliated Hospital of Zhengzhou University, Zhengzhou, China; ^2^ Department of Oncology, Hainan General Hospital, Hainan Affiliated Hospital of Hainan Medical University, Haikou, China; ^3^ State Key Laboratory of Esophageal Cancer Prevention and Treatment, the First Affiliated Hospital of Zhengzhou University, Zhengzhou, China

**Keywords:** high-grade B-cell lymphoma, high-intensity chemotherapy, prognosis, clinical features, treatment

## Abstract

**Objective:**

High-grade B-cell lymphoma (HGBL) is highly aggressive and has a poor prognosis.

**Methods:**

The clinical data of 76 patients with High-grade B-cell lymphoma treated in our lymphoma center from July 2016 to April 2020 were analyzed retrospectively. The clinical features, treatment and prognosis of patients with two types of high-grade B-cell lymphoma were compared and analyzed.

**Results:**

Among 76 patients with high-grade B-cell lymphoma, 44 cases (57.9%) were high-grade B-cell lymphoma, accompanied by MYC and Bcl-2 and/or Bcl-6 rearrangement (HGBLR) patients, and 32 cases (42.1%) were HGBL, NOS patients. The bone marrow infiltration, IPI (international prognostic index), Ann Arbor stage (III/IV), extranodal disease are more likely to occur in HGBLR group (P <0.05). Survival analysis of patients showed that overall survival (OS) and progression free survival (PFS) in HGBLR group were significantly shorter than those in HGBL, NOS group (median OS: 21 months vs not reached, P=0. 022; median PFS: 5 months vs 12 months, P = 0. 001). Further analysis demonstrated that, as compared with R-CHOP regimen, patients with HGBL who received high-intensity chemotherapy regimens (DA-EPOCH-R, R-CODOX-M/IVAC and R-Hyper-CVAD) had longer OS (median OS, 16 months vs not reached, P=0. 007) and PFS (median PFS, 5 months vs 11 months, P<0.001). Moreover, mu1tivariate ana1ysis showed that high-intensity chemotherapy regimens were independent risk factors for both PFS (P =0.001, HR: 0.306, 95% CI: 0.153–0.610) and OS (P =0.004, HR: 0.262, 95% CI: 0.105–0.656) in patients with HGBL.

**Conclusions:**

HGBLR patients have worse prognosis than patients with HGBL, NOS. High-intensity chemotherapy may improve the prognosis of patients with HGBL.

## Introduction

High-grade B-cell lymphoma (HGBL) was revised as an independent disease type in the 2016 new version of the WHO classification of hematopoietic and lymphoid neoplasm, which is highly aggressive and has a poor prognosis (1, 2). HGBL includes two types: high-grade B-cell lymphoma, accompanied by MYC and Bcl-2 and/or Bcl-6 rearrangement (HGBLR), and high-grade B-cell lymphoma, not otherwise specified (HGBL, NOS), this type has a low incidence and is clinically rare (1, 3–5). High-grade B-cell lymphoma (HGBL) with MYC and Bcl-2 and/or Bcl-6 rearrangements is an aggressive mature B-cell lymphoma that harbours a MYC rearrangement at chromosome 8q24 and a rearrangement in Bcl-2 (at chromosome 18q21) and/or in Bcl-6(at chromosome 3q27). These lymphomas are often called double-hit lymphomas, or triple-hit lymphomas if there are both Bcl-2 and Bcl-6 rearrangements in addition to the MYC rearrangement. The term “double-hit” as defined for this category refers only to the co-occurrence of MYC and Bcl-2 and/or Bcl-6 translocations. Lymphomas with two oncogenic translocations other than MYC (e.g. concomitant Bcl-2 and Bcl-6 translocations without a MYC breakpoint) or other gene translocations associated with MYC translocations (e.g. CCND1 translocations) are not included in this category. These lymphomas mainly occur in diffuse large B-cell lymphoma (DLBCL) and B-cell lymphoma, unclassifiable (with features intermediate between DLBCL and Burkitt lymphoma, BCLU), among which “double-hit” lymphomas are the most common (6–8). ”High-grade B-cell lymphoma (HGBL), NOS”, is a heterogeneous category of clinically aggressive mature B-cell lymphomas that lack MYC plus Bcl-2 and/or Bcl-6 rearrangements and do not fall into the category of diffuse large B-cell lymphoma (DLBCL), NOS, or Burkitt lymphoma (BL). However, they do share some morphological, immunophenotypic, and genetic features with these lymphomas. These cases are rare; the diagnosis should be made sparingly, and only when the pathologist is truly unable to confidently classify a case as DLBCL or BL.

At present, little is known about HGBL due to its rarity (9). And there is currently no standard treatment for HGBL. This study retrospectively analyzed the clinical data of HGBL patients (including HGBLR and HGBL, NOS), and compared the clinical features, treatment and prognosis of the two types of HGBL.

## Materials and methods

### Patients and data collection

This study was a retrospective analysis of 76 newly-diagnosed HGBL patients treated in our lymphoma center from July 2016 to June 2020. The inclusion criteria are as follows (1): pathologically, immunohistochemically and cytogenetic characteristics confirmed diagnosis of HGBL according to the WHO 2016 classification of the tumors and hematopoietic and lymphoid tissues (1), and was reviewed by at least two pathologists (2); with detailed treatment and follow-up data.

Data were retrospectively collected from medical records including Eastern Cooperative Oncology Group (ECOG), age, gender, involved sites, B symptoms, Ann Arbor staging, serum lactate dehydrogenase (LDH), serum β2-microglobulin (β2-MG), bone marrow involvement, Ki-67 level, International Prognostic Index (IPI), initial chemotherapy regimens were collected. The study was performed in accordance with the Declaration of Helsinki and was approved by the Medical Ethical Committee. The need for informed consent was waived by the ethics committee since only anonymised data were used for this retrospective study.

### Treatment and evaluation

Of the 44 patients diagnosed with HGBLR, 20 received first-line R-CHOP (rituximab, cyclophosphamide, doxorubicin, vincristine, and prednisone) chemotherapy and 24 were treated with a high-intensity chemotherapy which included DA-EPOCH-R (rituximab, dose-adjusted doxorubicin, cyclophosphamide, vincristine, etoposide, prednisone, n=15), R-CODOX-M/IVAC (rituximab, cyclophosphamide, vincristine, doxorubicin and methotrexate alternating with ifosfamide, etoposide, and cytarabine, n=4), and R-Hyper-CVAD (rituximab, hyper-fractionated cyclophosphamide, vincristine, doxorubicin alternating with cytarabine and methotrexate, n=5). Among all patients, the median number of induction chemotherapy cycles was six (range 1-10). Of the HGBLR patients, four patients received autologous hematopoietic stem cell transplantation (ASCT) and two received the chimeric antigen receptor T-cell immunotherapy after relapse.

Of the 32 patients diagnosed with HGBL, NOS, 15 received first-line R-CHOP chemotherapy and 17 were treated with a high-intensity chemotherapy which included DA-EPOCH-R (n=5), R-CODOX-M/IVAC (n=4) and R-Hyper-CVAD (n=8). Of the HGBL, NOS patients, three patients received an ASCT after complete remission and two patients received an ASCT after progressive disease. And high-dose methotrexate was added in four patients who presented central nervous system involvement. Because patients with HGBL are at higher risk of central nervous system involvement, we require intrathecal injection (IT) per cycle for patients with HGBL-R, those with a moderately high central nervous system invasion score, or those with central nervous system involvement on a regimen of cytarabine 50 mg, methotrexate 12 mg, and dexamethasone 5 mg. The above will be presented in **Table 1**.

**Table 1:** First-line treatment.

Regimens	HGBL	HGBL,R	HGBL ,NOS
	(n=76)	(n=44)	(n=32)
R-CHOP	35	20	15
High-intensity chemotherapy			
DA-EPOCH-R	20	15	5
R-CODOX-M/IVAC	8	4	4
R-Hyper CVAD	13	5	8
Consolidation therapy			
ASCT	8	3	5
allo-SCT	1	1	0
CAR-T	2	2	0

ASCT, Autologous Stem Cell Transplantation; allo-SCT, Allogeneic hematopoietic stem cell transplantation ; CAR-T, Chimeric Antigen Receptor T-Cell Immunotherapy.

Treatment responses were evaluated every two courses of chemotherapy. According to the International Working Group’s efficacy evaluation criteria (10, 11), the responses were divided into complete remission (CR), partial remission (PR), stable disease (SD), and progressive disease (PD), and (CR + PR) served as overall response rate (ORR). In the follow-up analysis, blood chemistries (including LDH, β2- MG, etc.) and imaging (including ultrasound, CT, MRI, or PET-CT) were performed every 3 months during the first 2 years, every 6 months until 5 years post-treatment, and then once yearly. Morphological examination of bone marrow cells was performed in patients with bone marrow infiltration at the beginning of disease.

### Follow-up and outcomes

The primary endpoints of this study were progression-free survival (PFS) and overall survival (OS). OS was defined as the time from the date of diagnosis to death from any cause or to the date of last follow-up. PFS was defined as time from initial diagnosis to date of progression, relapse, or death. The key secondary endpoints were the objective response rate (ORR) of the disease, which includes complete response (CR) and partial response (PR) evaluated by the physician and by central imaging review.

### Statistical analysis

Patient characteristics were assessed by descriptive statistical analysis. Medians and ranges are provided for continuous variables, and percentages are shown for categorical variables. Fisher’s exact test was used to compare categorical variables, and the Mann-Whitney U test and Kruskal-Wallis test were used to compare continuous variables. OS and PFS were estimated using the Kaplan-Meier method and were compared using the log-rank test. Multivariate analyses were carried out using the Cox proportional hazards model. The level of statistical significance was set to P < 0.05 for all analyses. All statistical analyses were two-sided and were performed using SPSS version 24.0 (IBM Corporation).

## Results

### Baseline characteristics

**Table 2** displays clinical characteristics and laboratory findings of the patients with HGBL. The median age was 47 years (range, 19–79 years) and 57.9% of patients (44/76) were male. 60.5% of patients (46/76) were in stage III-IV and 32.9% of patients (25/76) had B symptoms. In addition, 19 patients had bone marrow involvement and 42 patients had more than one extranodal site involved. The central nervous system was involved in 4 patients assessed by cytological examination of cerebrospinal fluid.

Comparisons of clinical characteristics in patients with HGBL, NOS and HGBLR were summarized in **Table 2**. There were 44 (57.9%) HGBLR cases and 32 (42.1%) HGBL, NOS cases. As compared to patients with HGBL, NOS, the HGBLR patients showed a higher Ann Arbor stage (P=0.002), bone marrow infiltration (P=0.032), more than one extranodal site involved (P=0.029) and higher IPI (P<0.001) at diagnosis. However, there was no statistical difference in the age, gender, B symptoms, ECOG score, LDH level, ki-67 level, and other clinical characteristics between the two groups.

**Table 2:** The comparison of clinical characteristics between the two groups.

Clinical characteristics		HGBL (n=76)	HGBL,R (n=44)	HGBL,NOS (n=32)	*P*
Age(year)	>60	22(29%)	16(36%)	6(19%)	0.095
	≤60	54(71%)	28(64%)	26(81%)	
Gender	male	44(58%)	24(55%)	20(63%)	0.488
	female	32(42%)	20(45%)	12(37%)	
ECOG score	>2	15(20%)	12(27%)	3(9%)	0.053
	≤2	61(80%)	32(73%)	29(91%)	
Ann-Arbor stage	I-II	30(39%)	11(25%)	19(59%)	0.002
	III-IV	46(61%)	33(75%)	13(41%)	
LDH(U/L)	<ULN	32(42%)	12(27%)	20(63%)	0.002
	≥ULN	44(58%)	32(73%)	12(37%)	
β2 -MG	<ULN	52(68%)	30(68%)	22(69%)	0.958
	≥ULN	24(32%)	14(32%)	10(31%)	
Bone marrow involvement	Yes	19(25%)	15(34%)	4(13%)	0.032
	No	57(75%)	29(66%)	28(87%)	
ESI	≤1	34(45%)	15(34%)	19(59%)	0.029
	>1	42(55%)	29(66%)	13(41%)	
B symptoms	Yes	25(33%)	17(39%)	8(25%)	0.212
	No	51(67%)	27(61%)	24(75%)	
Ki-67	<90%	31(41%)	16(36%)	15(47%)	0.357
	≥90%	45(59%)	28(64%)	17(53%)	
IPI score	≤2	35(46%)	11(25%)	24(75%)	0.006
	>2	41(54%)	33(75%)	8(25%)	
First-line therapy	R-CHOP	35(46%)	20(45%)	15(47%)	0.902
	High-intensity chemotherapy	41(54%)	24(55%)	17(53%)	

ECOG, Eastern Cooperative Oncology Group; LDH, lactate dehydrogenase level; β2-MG, beta-2 microglobulin level; ESI, extranodal sites involvement; ULN, upper limit of normal.

### Response to treatment

Among the patients who received R-CHOP regimen, 2 and 13 patient achieved CR and PR, respectively. SD was observed in one patient, and 19 patients achieved PD. Among the patients who received the high-intensity chemotherapy regimen, CR and PR were observed in 2 and 12 patients in DA-EPOCH-R group, respectively; 4 patient achieved CR and 2 patient achieved PR in R-CODOX-M/IVAC group; 9 patient achieved CR and 2 patients achieved PR in R-Hyper CVAD group (**Table 3**). The ORR of R-CHOP and the high-intensity chemotherapy regimen were 42.9 (15/35) and 75.6 (31/41), respectively (P=0.004).

**Table 3:** Responses to first-line treatment.

Regimens	Number of patients				
	CR	PR	SD	PD	ORR (%)
R-CHOP	2	13	1	19	42.9 (15/35)
DA-EPOCH-R	2	12	1	5	70.0 (14/20)
CODOX-M/IVAC	4	2	0	2	75.0 (6/8)
R-Hyper CVAD	9	2	0	2	84.6 (11/13)

CR, complete response; PD, progressive disease; PR, partial response; SD, stable disease; ORR, overall response rate.

Of the 44 HGBLR patients, 3 of them achieved complete response (CR) and 18 of them achieved a partial remission (PR), while stable disease (SD) was observed in 2 patients and progressive disease (PD) in 21 patients. The ORR of HGBLR patients was 47.7% (21/44). Among 32 HGBL, NOS patients, CR and PR was attained in 14 and 11 patients, respectively. PD was observed in 7 patients. The ORR of HGBL, NOS patients was 78.1% (25/32).

### Survival analysis and prognosis factors

**Figure 1:**
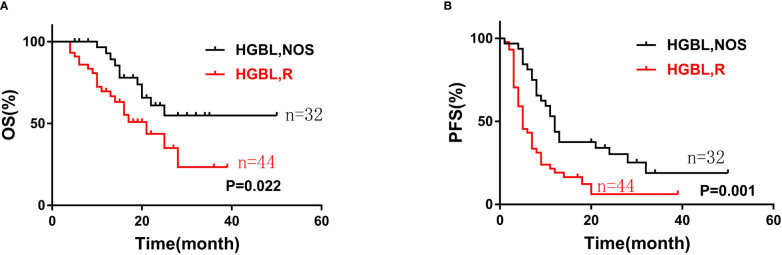
The overall survival **(A)** and progression-free survival **(B)** curves of patients with two types of high-grade B-cell lymphoma (HGBL): HGBLR (n=44) and HGBL, NOS (n=32).

At the end of the follow-up, 31 cases died, of which 2 patients died of adverse reactions after CAR-T cell immunotherapy. The median follow-up was 22 months (4 to 50 months). The 2-year OS rate of HGBL patients was 51.2% and the 2-year PFS rate was 45.3%. The HGBLR group have shorter OS and PFS than HGBL, NOS group (median OS: 21 months vs not reached, P=0. 022; median PFS: 5 months vs 12 months, P = 0. 001) (**Figure 1**).

**Figure 2:**
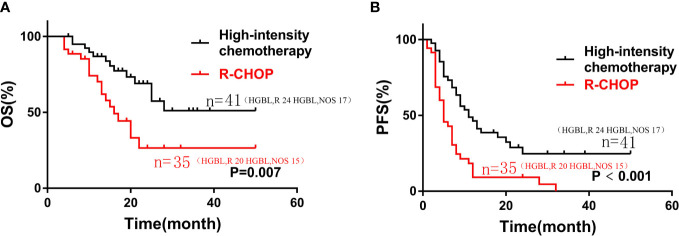
Kaplan-Meier curves of OS **(A)** and PFS **(B)** according to different induction chemotherapy regimen: high-intensity chemotherapy regimens (n=35) vs. R-CHOP (n=41) in patients with HGBL.

Compared with the patients who received the R-CHOP regimen, the patients who received the high-intensity chemotherapy (DA-EPOCH-R, R-CODOX-M/IVAC, or R-Hyper CVAD) had longer OS and PFS (median OS, 16 months vs not reached, P=0. 007; median PFS, 5 months vs 11 months, P<0.001), as shown in **Figure 2**.

**Figure 3:**
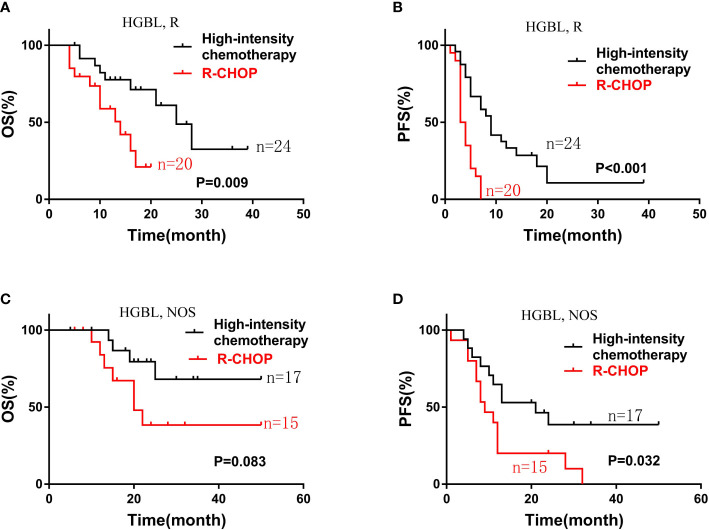
Kaplan-Meier curves of OS **(A)** and PFS **(B)** according to different induction chemotherapy regimen: high-intensity chemotherapy regimens (n=24) vs. R-CHOP (n=20) in patients with HGBLR. The OS **(C)** and PFS **(D)** curves of patients with HGBL, NOS stratified by different induction chemotherapy regimen: high-intensity chemotherapy regimens (n=17) vs. R-CHOP (n=15).

Subgroup analysis showed that patients with HGBLR who received high-intensity chemotherapy had longer OS and PFS (median OS, 25 vs 14 months, P=0. 009; median PFS, 9 vs 3.5 months, P<0.001) (**Figure 3**). Similarly, compared with HGBL, NOS patients treated with the R-CHOP regimen, patients treated with high-intensity chemotherapy had superior PFS (median PFS, 9 vs 21 months, P=0.032). Also, there was a trend toward a better OS for HGBL, NOS patients treated with high-intensity chemotherapy (P=0.083) (**Figure 3**).

Clinical features such as gender, age > 60 years, B symptoms, LDH > ULN, ECOG > 2, high Ann-Arbor stage, β2 -MG> ULN, extranodal sites involvement > 1, IPI > 2, bone marrow involvement, ki-67 and first-line therapy were included into univariate analysis. It demonstrated that age > 60 years (P = 0.003), B symptoms (P =0.020), ECOG > 2 (P = 0.007), β2 -MG> ULN (P = 0.006), bone marrow involvement (P = 0.013), extranodal sites involvement > 1 (P = 0.035), IPI > 2 (P = 0.001), as well as chemotherapy regimen (R-CHOP, P = 0.01) indicated shorter OS. Similarly, age > 60 years (P = 0.018), advanced Stage (III/IV) (P = 0.013), bone marrow involvement (P = 0.016), IPI > 2 (P=0.014) as well as chemotherapy regimen (R-CHOP, P < 0.001) were associated with shorter PFS (**Table 4**).

**Table 4:** Univariate analysis of risk factors associated with OS and PFS.

Characteristics	OS		PFS	
	HR (95% CI)	P value	HR (95% CI)	P value
Age (>60)	3.466(1.618-7.426)	0.003	2.528(1.403-4.554)	0.018
Gender	0.664(0.323-1.364)	0.253	0.910(0.525-1.577)	0.707
B symptoms	1.850(0.903-3.794)	0.02	1.137(0.651-1.985)	0.871
ECOG (>2)	2.159(0.982-4.747)	0.007	1.334(0.683-2.607)	0.737
LDH (>ULN)	1.201(0.571-2.527)	0.202	0.930(0.536-1.611)	0.171
Stage (III/IV)	1.476(0.689-3.161)	0.08	1.338(0.761-2.353)	0.013
β2 -MG (>ULN)	3.112(1.463-6.618)	0.006	2.392(1.305-4.387)	0.145
Bone marrow involvement	2.287(1.059-4.937)	0.013	1.658(0.893-3.079)	0.016
ESI>1	2.287(1.059-4.937)	0.035	1.264(0.731-2.188)	0.287
Ki-67≥90%	1.144(0.523-2.504)	0.667	0.723(0.414-1.260)	0.088
IPI score>2	2.569(1.220-5.409)	0.001	1.346(0.783-2.314)	0.014
High-intensity chemotherapy	0.551(0.266-1.141)	0.007	0.547(0.316-0.949)	< 0.001
HGBLR or HGBL, NOS	2.808(1.423-5.540)	0.022	2.788(1.556-4.998)	0.001

CI, confidence interval; HR, hazard ratio; OS, Overall survival; PFS, Progression-free survival; ECOG, Eastern Cooperative Oncology Group; LDH, lactate dehydrogenase level; β2-MG, beta-2 microglobulin level; ESI, extranodal sites involvement; IPI, international prognostic index; ULN, upper limit of normal.

Further, the multivariate analysis showed that advanced stage (III/IV) (P =0.011), β2 -MG> ULN (P =0.004), IPI > 2 (P = 0.015) and high-intensity chemotherapy (P =0.004, HR: 0.262, 95% CI: 0.105–0.656) were independent prognostic factors for OS. It was also found that high-intensity chemotherapy (P =0.001, HR: 0.306, 95% CI: 0.153–0.610) was independent prognostic factors for PFS (**Table 5**).

**Table 5:** Multivariate analysis of risk factors associated with OS and PFS.

Characteristics	OS		PFS	
	HR (95% CI)	P value	HR (95% CI)	P value
Age (>60)	1.132(0.448-2.862)	0.793	1.239(0.639-2.402)	0.526
B symptoms	1.234(0.468-3.258)	0.671	0.588(0.294-1.175)	0.132
ECOG (>2)	1.122(0.377-3.340)	0.837	0.996(0.403-2.464)	0.994
Stage (III/IV)	5.957(1.505-23.582)	0.011	0.929(0.406-2.125)	0.862
β2 -MG (>ULN)	3.692(1.508-9.039)	0.004	1.363(0.720-2.583)	0.342
bone marrow involvement	1.907(0.634-5.739)	0.251	1.284(0.582-2.831)	0.536
ESI>1	1.851(0.773-4.432)	0.167	1.154(0.619-2.152)	0.653
ki-67≥90%	0.766(0.327-1.793)	0.539	0.574(0.317-1.039)	0.067
IPI score>2	5.753(1.408-23.505)	0.015	1.590(0.758-3.337)	0.220
High-intensity chemotherapy	0.262(0.105-0.656)	0.004	0.306(0.153-0.610)	0.001
HGBLR or HGBL, NOS	3.147(1.301-7.611)	0.011	3.576(1.853-6.903)	<0.001

CI, confidence interval; HR, hazard ratio; OS, Overall survival; PFS, Progression-free survival; ECOG, Eastern Cooperative Oncology Group; ESI, extranodal sites involvement; β2-MG, beta-2 microglobulin level; IPI, international prognostic index; ULN, upper limit of normal.

## Discussion

High-grade B-cell lymphoma is a new category in the 2016 WHO classification and replaces the 2008 category (1). HGBL encompasses a group of rare and aggressive lymphomas. High morbidity and mortality of HGBL remain a major challenge (2, 12).

HGBLR has no special clinical manifestations, and is characterized by high aggressiveness, rapid progress, and poor prognosis (13, 14). Mainly occurs in the elderly, usually at an advanced stage (Ann-Arbor stage III/IV) at the time of diagnosis, often extranodal sites involvement, including bone marrow involvement, higher IPI scores and elevated LDH levels (15–20). In our retrospective study, HGBLR are more common in advanced stages (III/IV), with high IPI scores, involvement of extranodal sites, and higher LDH levels, which are consistent with previous literature reports (21). HGBL, NOS patients also have a highly aggressive clinical course. Due to the low incidence of this type, there is a lack of data to better describe its clinical characteristics (9). In our retrospective study, patients with HGBL, NOS are associated with lower Ann-Arbor stage, less bone marrow involvement, lower IPI score and lower LDH level.

Previous studies on B-cell lymphomas reported that the median survival time of HGBL was 4.5-34 months (22–29). In our study of 76 patients with HGBL, the median survival time was 25 months, which was consistent with previous literature reports. At a median follow-up of 22 months (4-50 months), the HGBLR group showed a significantly worse OS and a worse PFS than HGBL, NOS group, which is consistence with previous studies.

Currently, an international consensus standard treatment has not been established for HGBL. Multiple retrospective studies of R-CHOP have shown a worse outcome in patients with MYC rearrangement than in patients without MYC rearrangement, and suggest improved outcomes after more intensive treatment (30, 31). A retrospective study showed that patients with double-hit lymphoma may be may benefit from intensive induction (29). However, another recent retrospective study demonstrated that the first-line treatment (R-CHOP compared with intensive chemotherapy) was not significantly associated with OS in double-hit and triple-hit lymphomas (32). In our retrospective study of 44 HGBLR patients, 20 (45.5%) received first-line R-CHOP treatment, and 24 (54.5%) received first-line high-intensity chemotherapy. Of the 32 patients with HGBL, NOS, 15 (46. 9%) received R-CHOP as the first-line treatment, and 17 (53. 1%) received high-intensity chemotherapy as the first-line. It demonstrated that, compared with HGBL patients who received R-CHOP, patients who received high-intensity chemotherapy had significant longer OS and PFS. Further multivariate analysis showed that the high-intensity chemotherapy was an independent risk factor for better prognosis in patients with HGBL. Moreover, we conducted a subgroup analysis of HGBLR and HGBL, NOS patients receiving different intensity chemotherapy regimens. In HGBLR patients, compared with patients receiving R-CHOP regimen, patients receiving high-intensity chemotherapy had longer OS and PFS. Similar results were obtained in HGBL, NOS patients. HGBL patients who received hematopoietic stem cell transplantation may have better prognosis and treatment response rates than those who did not receive hematopoietic stem cell transplantation, but the difference was not statistically significant. It is indicated that large-scale multicenter clinical studies are still needed to further determine the therapeutic role of hematopoietic stem cell transplantation in HGBL patients.

There are several limitations in present study. First, the sample size of 76 was relatively small. However, considering the extreme rarity of HGBL, the results of our study are worth consideration. A prospective, large-sample, multi-center study is required for further investigation. Second, as a retrospective study, patients were not randomly assigned to high-intensity chemotherapy versus R-CHOP, which meant that the choice of high-intensity chemotherapy or not might have been biased by the treating physician’s preference based on patient’s characteristics. Thus, even though high-intensity chemotherapy was found to be a prognostic factor for survival in our study, it is important to reemphasize the potential bias in patient selectivity undergoing high-intensity chemotherapy, so this should require validation in a larger cohort in the future.

In conclusion, HGBL is a highly aggressive, rapidly progressing lymphoma with no standard chemotherapy regimen. This study demonstrated that HGBLR has a worse prognosis than HGBL, NOS, and high-intensity chemotherapy was an independent risk factor predicting longer OS and PFS in patients with HGBL.

## Data availability statement

The original contributions presented in the study are included in the article/supplementary material. Further inquiries can be directed to the corresponding authors.

## Ethics statement

The studies involving human participants were reviewed and approved by Ethics Committee of the First Affiliated Hospital of Zhengzhou University. Written informed consent from the participants’ legal guardian/next of kin was not required to participate in this study in accordance with the national legislation and the institutional requirements.

## Author contributions

ZML and MZZ designed the research and was the chief person in charge of the manuscript. YFC, QC and YC performed the material preparation and data collection. YFC, QC and YC contributed equally to this work. Data analysis was performed by YFC, QC, YC, ZML and MZZ. The manuscript was written by YFC, QC, YC, ZML and MZZ. All authors contributed to the article and approved the submitted version.
